# A communication hub for a decentralized collaboration on studying real-life cognition

**DOI:** 10.12688/f1000research.6229.1

**Published:** 2015-03-10

**Authors:** Michael Hanke, Yaroslav O. Halchenko

**Affiliations:** 1 Department of Psychology, University of Magdeburg, Magdeburg, Germany; 2 Center for Behavioral Brain Sciences, Magdeburg, Germany; 3 Department of Psychological and Brain Sciences, Dartmouth College, Hanover, NH, USA; 4 INCF Data-sharing taskforce, Karolinska Institute, Stockholm, Sweden

**Keywords:** fMRI, rich natural environment, Quasi-naturalistic stimulation

## Abstract

Studying the brain’s behavior in situations of real-life complexity is crucial for an understanding of brain function as a whole. However, methodological difficulties and a general lack of public resources are hindering scientific progress in this domain. This channel will serve as a communication hub to collect relevant resources and curate knowledge about working paradigms, available resources, and analysis techniques.

## Editorial

Everyday cognition involves a large variety of concurrent neural processes that handle an incredible amount of sensory inputs in order to generate appropriate responses when interacting with the environment. It can be argued that studying any of these aspects of cognition in isolation, as it is often the case in feature-deprived laboratory experiments, yields an over-simplified or over-specialized understanding of the true nature of brain function. In order to fully understand “how the brain works”, it is essential to study the complex inter-play of cognitive processes in a rich natural environment and go beyond the localization of individual aspects of brain function.

The increasing number of publications focused on multivariate and data-driven analysis of brain network dynamics is a clear indication that researchers embrace this challenge, on both a conceptual and a methodological level. At present, most studies in this domain use the “resting state” paradigm ^1^, where spontaneous activity of brains at rest (i.e. not performing any particular uniform task) is recorded. However, this paradigm has limited utility for studying how we process information about the complex environment that surrounds us, as resting-state measurements provide little information about the driving forces behind the observed activity patterns.

Quasi-naturalistic stimulation (e.g. a movie) is the second most used paradigm and enables studies of the dynamics of neural processes across multiple individuals in contexts similar to real life. Movies, as rich, time-locked stimuli, have played a central role in studies on long-term memory performance ^2^, functional brain parcellation ^3, 4^, functional alignment ^5^, temporal ^6^ and multisensory information integration ^7^, emotion ^8, 9^, as well as identification of homologous brain areas across species ^10^. While there is some evidence that movie stimuli are a promising approach to study the properties of brain areas that typically exhibit little response modulation in traditional experimental paradigms ^11^ and that they, in comparison to resting-state data, provide a different perspective on aspects of the functional organization of the brain, such as interregional connectivity ^12^, it is unclear to what degree watching movies actually resembles natural viewing conditions ^13, 14^.

Beyond pre-recorded naturalistic stimuli, virtual reality environments ^15^ and data acquisition outside the laboratory ^16^ allow for the observation of brain activity during interaction with a rich environment, as opposed to the more passive processing of sensory inputs. However, with the increasing complexity of the stimulation, it also becomes more difficult to understand which stimulus properties are driving particular patterns of brain activity. Consequently, an in-depth understanding of the nature of a stimulus is of paramount importance for the interpretation of statistical properties of brain activity ^17^, and can enable more detailed analysis, even in the context of data-driven methods ^18^.

Together, these methodological difficulties, unavoidable confounds, and a comparably large amount of noise are likely the contributing factors to the current state where only a small fraction of the literature is concerned with aspects of complex everyday cognition. In our opinion there are two main challenges that will determine the success of research on real-life cognition in terms of both quality and quantity of scientific output: the availability of adequate datasets and the development and evaluation of analysis methods capable of disentangling the complex mixture of reflections from multiple concurrent neural processes. In some ways this represents a classic chicken-and-egg problem as the lack of datasets inhibits the development of methods, and the lack of suitable methods makes the collection of adequate datasets a risky and expensive endeavour.

### Share materials, successes, and failures

The purpose of this forum is to cut this Gordian knot by documenting applicable paradigms, available resources, and scientific findings. It aims to serve as a platform for an interdisciplinary exchange of ideas to move us closer to an understanding of the function of the brain as a whole, outside of conventional feature-deprived laboratory settings. Unlike more traditional publication channels, F1000Research offers two major advantages in this regard: a) almost instantaneous publication promises much lower latency for community interactions; b) no threshold on the significance of a report. The latter aspect is especially important, as in any situation of uncertainty it is just as important to know what works as it is to know what is particularly challenging.

Consequently, this channel is open to a wide range of contributions. This includes descriptions of available resources (e.g. natural stimuli in the form of images, sounds, movies, or virtual environments) to study cognition in real-life situations inside and outside the laboratory. Equally important are studies that evaluate the utility of particular analysis methods on these data, such as comparative benchmarks but also (failed) replications of previous studies. We want to put a particular focus on small scale projects that often end up in a lab drawer. With increasing availability of versatile datasets (such as our Forrest Gump movie brain imaging project ^19^), we anticipate more and more “feasibility” studies that explore the applicability of natural stimulation paradigms for particular aspects of cognition. We explicitly encourage such publications using the articles types Data Note and Research Note.

We hope that this channel will serve as a hub for a decentralized, inter-disciplinary collaboration that facilitates studies of everyday cognition. Track and contribute to this channel to stay in touch with the latest developments and contributions.
